# Does the Carer Support Needs Assessment Tool (CSNAT) cover the support needs of young carers? A systematic literature search and narrative review

**DOI:** 10.1177/02692163251363476

**Published:** 2025-08-25

**Authors:** Yuen Ki Fung, A Carole Gardener, Morag Farquhar

**Affiliations:** School of Health Sciences, University of East Anglia, Norwich, UK

**Keywords:** Caregivers, child, systematic review

## Abstract

**Background:**

Young carers support family members with a range of health issues but with known detrimental impacts on young carers themselves suggesting they require support. The Carer Support Needs Assessment Tool (v3.0) is a well-established tool enabling unpaid/family carers to identify and express their support needs to healthcare professionals. However its comprehensiveness for young carers is unknown.

**Aim:**

To explore whether the Carer Support Needs Assessment Tool (v3.0) covers the support needs of young carers identified within published literature.

**Design:**

Systematic literature search and narrative review. English language studies were identified against predetermined inclusion/exclusion criteria through searching databases and reference lists of included papers. Papers were critically appraised, and data extracted and synthesised by three reviewers. Identified needs were mapped to the Carer Support Needs Assessment Tool (v3.0) questions.

**Data sources:**

CINAHL (EBSCO), EMBASE (Ovid), Applied Social Science Index and Abstract, Medline (EBSCO), American Psychological Association, PsycINFO and the Cochrane Database of Systematic Reviews (January 2010–December 2024).

**Result:**

Thirty-four papers were included. Synthesis of findings confirmed that young carers have (often unmet) support needs relating to information, emotional distress, relationships (including parent-child relationships), accessing services and education. Mapping these to the Carer Support Needs Assessment Tool (v3.0) questions suggested it requires adapting to ensure coverage of education and parent-child relationships.

**Conclusion:**

Young carers can require support across many areas, suggesting they would benefit from identifying and expressing their needs to healthcare professionals. The Carer Support Needs Assessment Tool Intervention could enable this but requires identified adaptations to the v3.0 tool.

**What is already known about this topic?**

- Young carers play a vital role in supporting family members with a disability, physical illness, mental health condition or drug or alcohol problem.
- Young carers may have unidentified support needs that could be a target for intervention.
- The Carer Support Needs Assessment Tool (CSNAT) is an evidence-based, person-centred tool to enable identification and expression of adult carer support needs, however its comprehensiveness for young carers is unknown.

**What this paper adds?**

- Synthesis of knowledge relating to young carer support needs from the published literature, including needs carers felt were met or unmet, and inputs carers considered helpful.
- Mapping these support needs to the CSNAT (v3.0) identified that young carers’ educational needs and parent-child relationships were not adequately covered by the tool, suggesting that adaptions could be made to enhance its comprehensiveness for young carers.

**Implications for practice, theory or policy**

- Many of the support needs of young carers are unmet: particular areas of concern relate to information needs, emotional distress, relationship issues, access to services and education needs.
- Young carers would benefit from identification, and from a comprehensive, holistic, person-centred assessment of their needs and appropriate response to these needs.
- The CSNAT holds promise for identification of unmet young carer support needs although it would benefit from amendment to ensure it encompasses the full range of potential needs of this group.

## Introduction

Young carers have been defined as those under 18 years who help look after a relative with a disability, illness, mental health condition or drug or alcohol problem.^1^ It is currently estimated that around 7%–8% of children in the UK and Europe are young carers^2^ and that there are similar numbers in the USA, Canada and Australia.^3,4^ The Commonwealth Charter for Young Carers has also highlighted the presence of young carers worldwide.^5^ However, inconsistencies in definitions used, the lack of up-to-date statistics in many countries, combined with the reluctance of some young people to identify as a carer suggests that the total global number of young carers remains unknown.^2,3,6^

Existing evidence has shown that young carers carry out household tasks, provide emotional and physical support, look after younger siblings, administer medication, manage and support household finances, provide mobility assistance and act as a communication facilitator for the care recipient.^2–4,7^ However, it is also well documented that being a young carer can result in highly detrimental impacts on mental health, physical health and well-being.^4,8–15^ Studies have shown that the role is associated with high levels of stress, anxiety and depression.^12^ There is evidence that young carers are more likely to report physical health problems than their peers, and that being a young carer is linked to higher mortality rates.^8,12,16,17^ There are also known detrimental impacts on education, employment opportunities and financial security.^15,18,19^ Young carers themselves describe having little time to follow their own interests,^20^ and feel they are often seen primarily as a carer rather than a child with a full life ahead of them.^5^ Young carers, and organisations who represent them, have highlighted the need for greater support to mitigate these impacts.^5,19,21^

In the UK, statutory authorities have a responsibility to assess the support needs of young carers and consider preventative strategies in relation to their caring role,^6^ but young carers can be reluctant to participate in assessments for fear of being brought to the attention of social services. Healthcare professionals have a role to play in assessing and responding to the impact of being a young carer (alongside colleagues in social care, education and the voluntary sector)^6^ by opening up vital, but less intimidating, conversations with young carers about support they may need. Existing tools and interventions to facilitate this currently focus on young carer identification and understanding the context and impact of the role on a young carer’s life.^22^ There is also a growing awareness of the need to consider a whole family approach.^4,6^ However there remains a lack of evidence-based interventions that are purposively designed to pro-actively involve young carers in conversations with healthcare professionals about their support needs and the actions required to address them.

The Carer Support Needs Assessment Tool Intervention (CSNAT-I) is a well-established evidence-based means to enable unpaid/family carers to identify and express their support needs to healthcare professionals and enable tailored support.^23–26^ CSNAT-I is a comprehensive, person-centred intervention facilitating holistic support to unpaid/family carers and has been adopted internationally for adult carers: https://csnat.org.^27–29^ It comprises two components: (1) a carer-completed evidence-based, 15 question tool that comprehensively encompasses carers’ holistic support needs (CSNAT v3.0), which is integrated into (2) a five-stage person-centred process that facilitates assessment, response and review of carers’ support needs.^29^ However, the CSNAT^27,28^ – the tool itself – was origionally developed and validated only with adult carers and so the relevance of its questions to young carers, and therefore its potential utility for young carers, is unknown. In conducting a narrative review to establish the comprehensiveness of an earlier version of the CSNAT (CSNAT v2.0) for adult carers of patients with chronic obstructive pulmonary disease (COPD), Micklewright and Farquhar^30^ noted that the needs of young carers were likely to vary significantly from adult carers and recommended that a dedicated review was required to inform a specialist support needs assessment tailored for this age group (see Supplemental Material Appendix B Inclusion Criteria).^31^

This review therefore aims to systematically identify the support needs of young carers from the published literature and use the findings to examine the comprehensiveness of CSNAT v3.0 for young carers through question mapping.

## Methods

### Review question

Does the CSNAT (v3.0) comprehensively cover the published support needs of young carers?

### Study design

A systematic search of peer-reviewed English language research literature published between 2010 to 2020, and subsequently updated to 2024, was conducted by three reviewers, informed by scoping searches. The data extracted were synthesised and mapped to the CSNAT v3.0 questions, then narratively described. This pragmatic approach was considered the most appropriate for the review question and was methodologically guided by Micklewright and Farquhar’s^30^ previous CSNAT-related review and mapping exercise for adult COPD carers. The initial young carers’ review protocol (2010–2020) was registered (Prospero database: CRD42021238883, available at https://www.crd.york.ac.uk/prospero/display_record.php?ID=CRD42021238883); no other post-registration changes were made to the protocol beyond extending the search to 2024. The PRISMA checklist and flowchart was used to ensure clarity in reporting of activity at the various stages of the review.^32^ The required licence granting use of the CSNAT v3.0 for this review was obtained.

### Study identification

A scoping search identified that young carers had a range of support needs across physical, mental and social domains, emphasising the need to incorporate a multi-disciplinary perspective into the literature search. Relevant papers were therefore sought by searching a range of electronic databases including CINAHL (EBSCO), EMBASE (Ovid), Applied Social Science Index and Abstract, Medline (EBSCO), American Psychological Association, PsycINFO and the Cochrane Database of Systematic Reviews. Grey literature was not included, however reference searching of included studies supplemented the search strategy.

The search terms are outlined in Table 1. These were informed by the previous CSNAT-related review for COPD carers^30^ in which search terms were chosen to reflect variation in how unpaid/family carers are referred to, both by themselves and by others. Where appropriate potential search terms were adapted for young carers.^30^

**Table 1.** Search terms.

Search terms				
Age group ‘OR’ between terms		Population ‘OR’ between terms		Interest ‘OR’ between terms
child* OR adolescent* OR youth* OR teenager* OR young*	AND	carer* OR caregiver* OR supporter* OR informal* OR famil* OR friend* OR relative* OR lay*	AND	support* OR need*
**Expanders:** similar terms				
**Limiters:** 2010–2024, English language, peer-reviewed, qualitative studies, young carers who are aged 18 years or under				
Search terms limited to titles only				

The inclusion and exclusion criteria are outlined in Table 2. Eligible studies had to include participants who were children (aged 18 years or under), who provided unpaid care for people with any type of health condition (physical/mental). Again informed by the previous CSNAT-related review for COPD carers,^30^ and by CSNAT development work,^27^ relevant papers also had to include information about the young carers’ support needs and/or ‘helpful inputs’: that is, inputs they considered a helpful response to their needs. Editorials, opinion pieces, case studies and non-empirical studies were excluded.

**Table 2.** Inclusion and exclusion criteria with key justifications.

Inclusion criteria	Justification
Papers that included information about the support needs of children who are unpaid/family carers of patients with any health condtion (physical/mental)	To ensure the included papers contained findings relating to the review question
Qualitative studies	To ensure extraction of support needs identified by young carers themselves
Unpaid/family carers who are aged 18 years or under	Some definitions of young carers include those aged 18 years, but some state under 18 years (e.g. NHS 2021), however the age of 18 years is considered a transition year from child to adult Schraeder et al.^33^ and so is included
Peer-reviewed papers only	To increase confidence in the quality of the papers
Papers written in English	No translation resources available
Publishing period: January 2010 to December 2024	To ensure data relating to the contemporary support needs of young carers
Exclusion criteria	Justification
Paid/professional carer (those who have healthcare related qualification or provide paid care work)	Paid/professional carers are likely to have different support needs compared to (young) unpaid/family carers who are the focus of the review question
Editorials, expert opinions and case studies	Lower quality evidence was excluded to increase the reliability and strength of the findings

Titles and abstracts of potentially relevant papers were screened by a first reviewer (initial search YKF; updated search CG) against the eligibility criteria. If eligibility remained unclear a full text reading was undertaken. In cases of on-going uncertainty, the text was discussed with the second reviewer (MF) to ensure a consensus for both searches. The second reviewer (MF) also reviewed 10% of all identified papers independently to establish reproducibility of screening for both searches (agreement was high).

### Quality assessment

An assessment of all included papers was carried out to establish methodological quality. As per the inclusion criteria, all identified papers reported on qualitative studies therefore quality assessment was undertaken using the Critical Appraisal Skills Programme (CASP) Checklist for qualitative papers.^34^ The quality assessment was completed by a first reviewer (initial search YKF; updated search CG), and four studies (11%: two from each search) were randomly selected for independent appraisal by the second reviewer (MF); the assessments were then compared to establish accuracy (agreement was high) and ensure the consistency in quality assessment.

### Data extraction

In line with the previous CSNAT-related review for COPD carers’ methods,^30^ data on young carers’ support needs were identified from the included papers then extracted and grouped using Ewing and Grande’s^27^ framework of three types of carer support need data: their (1) unmet needs, (2) met needs and (3) helpful inputs. In the context of young carers, ‘unmet needs’ were support needs that young carers considered unresolved or unsupported, whereas ‘met needs’ were support needs that young carers considered to be resolved, potentially via intervention from health, social or other services. ‘Helpful inputs’ referred to the support or responses that young carers identified as helpful in addressing, reducing or resolving their support needs. The concept of ‘support needs’ was based on Bradshaw’s concept of ‘expressed need’^35^: this is in keeping with the role of the CSNAT as a tool used to identify the needs of a unique individual through self-report.

Using this framework two reviewers initially, but independently, extracted data from five eligible papers (15%: three from initial search; two from updated search). Following comparison of the reviewers’ findings (agreement was high), a consistent approach to the extraction and grouping of data was established. The first reviewer (initial search YKF; updated search MF) subsequently extracted the relevant data from the remaining papers.

### Data analysis

The different types of support needs extracted from included papers (unmet needs, met needs and helpful inputs) were then mapped to the 15 CSNAT v3.0 questions. To do this two reviewers examined each support need identified from the young carers’ literature and, in turn, considered them in relation to each of the 15 CSNAT v3.0 questions to identify whether, and to which question(s), the need could be mapped. In line with the principles established by the original developers of the CSNAT, this process allowed for several different support needs to be mapped to the same CSNAT v3.0 questions (reflecting the domain-based nature of the CSNAT questions),^27^ and for some individual support needs to be mapped to more than one CSNAT v3.0 question. This is in keeping with how the tool functions in practice.

Identified support needs that could not be mapped onto the tool’s existing questions suggested areas where amendements to the tool might be necessary to ensure comprehensiveness for young carers. These unmapped support needs were reviewed to establish whether they could be incorporated into existing questions on the tool (i.e. through modification of question wording) or, following the approach of the previous CSNAT-related review for COPD carers,^30^ synthesised to represent one or more additional broad areas of support need or new ‘domains of need’ (i.e. new questions on the tool). This process was achieved through discussion within the research team (which included one of the developers of CSNAT v3.0: MF), followed by consultation with one of the two original CSNAT developers.

Once agreement was reached about amendements required to improve comprehensiveness for young carers, a narrative description of the findings was produced. As noted by the previous CSNAT-related review for COPD carers,^30^ this analytic approach was congruent with the paradigm of pragmatism in providing the best methodological approach to answer the very applied review question.^36^

## Results

### Study selection

**Figure 1.**
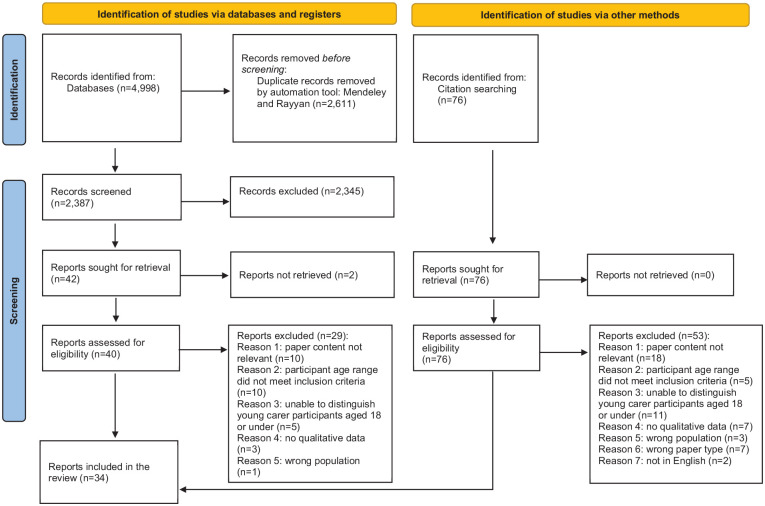
Prisma flow chart (Page et al.^32^).

The searches yielded 34 eligible papers (see Figure 1): all were qualitative and their characteristics are reported in Appendix 1 (Supplemental Material). Included papers reported findings from the UK,^37–49^ Canada/USA,^50–54^ Australia,^55–58^ Norway,^59,60^ Italy,^61,62^ Slovenia,^62^ Germany,^63^ Austria,^64^ Switzerland,^65^ Belgium,^66^ Tiawan,^67^ South Africa^68^ and West Kenya.^69^ Study participants were recruited via community advertising and a range of health, community and educational organisations. The age of young carers across all included studies ranged between 6 and 18 years. Nine studies also included data relating to young adult carers, however data were only extracted for the carers aged 18 years or younger.^41,43,47,49,55,57,58,65,68^ Five studies reported data from adults looking back on their time as young carers.^42,50,51,54,67^

### Quality appraisal

The quality appraisal findings reported in Appendix 2 (Supplemental Material) highlighted a number of issues that may have impacted the validity of the findings reported in the papers. Some studies chose an indirect approach to exploring young carers’ experiences such as (as noted) conducting retrospective interviews with adults about their experiences of being a young carer,^42,50,51,54,67^ or by using vignettes.^39^ One study collected data from young carers in the presence of their care recipients which may have censored how young carers spoke about their caregiving experiences.^66^ Some studies provided limited explanations as to how relevant data were retrieved from interviews and the themes developed.^50,52,55^

### Overview of findings relating to identified young carers’ needs

The literature reviewed identified that young carers have a range of support needs, many of which are unmet. In particular, young carers require support in relation to information needs, emotional distress, relationship issues, access to support services and education. These five key areas of need are outlined below, before the mapping of young carers’ support needs to the CSNAT v3.0 questions is presented.

#### Information needs

Having information and knowledge about the person being cared for was seen as important for young carers, but was an area where they frequently failed to receive the support needed. Many of the included papers noted young carers lacked access to information about the cared-for person’s illness and their care (e.g. McAndrew et al.,^37^ Blake-Holmes and Cook,^42^ Phelps,^48^ Stevens et al.,^49^ Nichols et al.,^53^ Mauseth and Hjälmhult,^59^ Trondsen,^60^ D’Amen et al.,^61^ Santini et al.,^62^ Leu et al.,^65^ Van Parys and Rober^66^ and McGibbon et al.^70^). As a result, young carers often obtained information indirectly, such as by overhearing household conversations, leading them to interpret these on their own.^38^

Even when information was discussed directly with them, young carers reported receiving inconsistent and vague information which could cause confusion.^38,66^ Similarly, some stated that clinicians failed to provide disease-related information, did not update them on their parents’ condition or used professional language rendering communication ineffective.^37,38,53^ In response young carers highlighted the need for an individualised approach, including use of age-appropriate methods for information delivery.^38,53,59^

#### Emotional distress

Many of the young carers described unmet needs in relation to their fears and worries. For some this arose from feelings of uncertainty in relation to the cared for person’s illness.^38,60,66,67^ Here young carers described worrying about symptom management, changes in the cared-for person’s health status and uncertainty about the future. Young carers also described the distress experienced in response to perceived stigma from their peers associated with both with their role^40,56^ and with the presence and nature of illness in their family.^38,51,52,59,63^ Finally, the dual roles of being a child (with school work) and a carer (with responsibilites at home) could result in exhaustion and feeling overwhelmed.^37,46,52,55,60,65^ Family members and healthcare profesisonals were identified as key sources of support for this (e.g. Svanberg et al.,^40^ Newman et al.,^51^ Nichols et al.^53^ and Trondsen^60^). Where young people didn’t have access to someone with whom they could discuss these feelings their distress was often compounded by a percieved need to disguise and self-manage their emotions, and focus instead on the needs of the person they supported.^59^

#### Relationship issues

Young carers also identified unmet needs in relation to interperonal relationships, including challenges in relationships with peers, healthcare professionals and family. Many of the included papers mentioned the time cost of caregiving that led to frequent absence of young carers from social activities, and subsequent feelings of social isolation (e.g. McAndrew et al.,^37^ O’Dell et al.,^39^ Svanberg et al.,^40^ Charles et al.,^50^ Stamatopoulos,^52^ Hamilton and Adamson,^55^ Moore et al.,^56^ Mauseth and Hjälmhult^59^ and Leu et al.^65^). Difficulties establishing supportive relationships with healthcare professionals were similarly highlighted.^37,38,51,53,60^

Family relationships were also affected. Young carers felt dissatisfied with the lack of recognition from parents in relation to their caregiving contribution^40,59^ and the unequal division of caring responsibilities within the family.^52^ Family dynamics could also be impacted by deterioration in a sick parent’s behaviour or personality, leading in some cases to family breakdown.^38,52,56^ Young carers also described having to take on additional roles caring for siblings^56^ and the need for practical support to assist with this, for example, help with school pickups.^37^ Some studies noted the financial stress on the whole family, for example, from loss of income when the parent was not working, or in response to funding addictions and the subsequent need for more financial stability.^37,43,48,49,56,59,61,62,69^ A key finding was that being a young carer to a parent could lead to loss of the parent-child relationship: ^37–40,42–44,50–53,56,59–61,68,71^ this particular area of need is discussed further below, under ‘Mapping needs to CSNAT questions’.

#### Access to services

Challenges in access to support services were raised in some of the included papers.^40,49,61,62^ The difficulties experienced by some young carers in obtaining access to training and support, and the infrequency of that support, was noted.^40^ Some had no knowledge of the available services for young carers^37^: poor advertising of support services and the different definitions of ‘young carers’ in agencies were noted.^51^ Some young carers welcomed the idea of distant support using technology, although in-person support was preferred.^53^ There was a particular unmet need around knowing what financial support was available, and how to access it.^43,49,61,62^ The lack of available family support for immigrant families was highlighted, resulting in feelings of isolation for these particular young carers.^50^

#### Education needs

Support needs related to education were commonly discussed in the papers. Given the age range of young carers, education had an important place in their daily lives but caring responsibilities led to negative impacts on education such as absenteeism, attention loss in class and reduced learning ability.^38,40,46,49,52,54,57,60,65,67–69^ Future education plans were also impacted by caring responsibilities. Two studies noted that young carers were particularly knowledgeable about universities’ locations and course workloads, such that they could maintain a balance between studies and caring duties.^52,55^ Studying abroad became a difficult decision for young carers to make.^59^ In some extreme cases, young carers chose to postpone their further education to fulfill the young carer role.^52^ Again, this particular key area of need is discussed further below, under ‘Mapping needs to CSNAT questions’.

### Mapping needs to CSNAT questions

CSNAT v3.0 was found to cover most of the young carers’ support needs identified from the review, and no CSNAT questions appeared to be redundant for young carers. However, support needs related to education issues and parent-child relationships were two areas in particular that some young carers required support with but which were not, or not adequately (given their particular importance to the young carers’ age group), covered by CSNAT v3.0. Data informing the mapping exercise is reported in Appendix 3 (Supplemental Material).

First, the educational needs of young carers requires attention. As noted, the included papers documented how the caregiving role affected young carers’ ability to fulfill their student obligations and plan for their future education. Constructive suggestions for addressing these issues included partnering young carers with school teachers and nurses to develop a ‘checklist’ on the needs of young carers, and flexible handling of young carers’ attendance and schoolwork submission dates.^37^ Enhancing teacher awareness of young carers and their needs was also noted.^41,43,49,57,63^ The reported positive effect that accessing education could have as a source of support and information^37,38,41,43,49,51,57,63,65,66,68,69^ and distraction from the caring role^54,64,68^ is also worthy of note.

Second, the loss of the parent-child relationship was identified as an area of support need for young carers not adequately covered by the CSNAT v3.0. Missing out on a ‘normal’ parent-child relationship due to the health condition of the sick parent and the caring role was discussed in many of the reviewed papers. For example, for young carers of parents with dementia, the quality of interactions was diminished, leading to frustration with the parent-child relationship.^53^ Further, a young carer with their own chronic condition could not count on their dependent parent to provide care for her, which led to feelings of loss of love in the filial relationship.^51^ Some young carers shared the experiences of being bullied in school but not being able to rely on sick parents to manage this, making them feel helpless.^37^

Young carers also compared their parent-child relationships with those of their peers: some were jealous of their peers who had non-dysfunctional relationships with their parents and felt frustrated with their own poor parent-child relationship.^51,60^ This parent-child relationship loss led to low self-esteem, despondency, poor academic performance and poor peer relationships.^37,40,56^ The reviewed papers reported that some young carers’ parents were not capable of performing expected parenting responsibilities, such as providing ‘emotional warmth’, ‘stability’ and ‘guidance’ to meet young carers’ developmental needs.^37,39,50,51,56,59,60^ Thus, these parents were seen as unreliable, leading to the feeling of powerlessness in some young carers.^40^ Some young carers sought ‘pseudo parental support’ from friends^52^ and one young carer reported learning to be self-sufficient to deal with the challenges that she had in life instead of relying on her parents.^39^

## Discussion

### Main findings/results of the study

This review aimed to investigate the comprehensiveness of CSNAT v3.0 for identifying the support needs of young unpaid/family carers. The review process first identified young carers’ support needs (areas where young carers experience a range of challenges due to their caring role and perceived shortfalls in support available), before using the findings to explore CSNAT v3.0’s comprehensiveness for young carers. The very young age of some of the carers (some as young as 6 years old) was notable but in line with national reports.^7^

#### Unmet needs experienced by young carers

Unmet needs experienced by young carers included accessing information, support with emotional distress, managing relationships (including parent-child relationships), access to services and educational support.

The review highlighted the need to increase information sharing with young carers, and to consider ways to deliver this information effectively. Young carers noted that they lacked information about the nature of their parent’s illness and how to manage symptoms and care. Furthermore they described how, even when they were included in information sharing processes, the information was often presented in a way that wasn’t accessible to them. Young carers also expressed how this lack of information left them vulnerable to speculation, mis-information and feelings of concern. There is therefore a need for healthcare professionals to ensure that young people are included in the information transfer process and to adopt an individualised approach in terms of how the information is presented; this supports findings in the wider literature.^13,72^

Professionals can also make a difference supporting young carers’ emotional well-being. Studies within the review found that young carers can feel emotional strain in response to worries about the cared-for person, the direct demands of their caring role and the resulting strain that the role puts on other aspects of their lives. These findings are in line with quantitative studies that suggest young carers have poorer mental health than their non-caregiving peers.^12^ In response, young carers described how they valued the opportunity to discuss their feelings and worries with family members, healthcare professionals and counsellors. Healthcare professionals can therefore play a role either in directly providing emotional support or enabling access to well-being services.

Young carers also described needing support to maintain and develop relationships with peers, healthcare professionals and within their families. In particular, the review found that young carers often need support to manage family relationships where they can require help to negotiate the nature of their caring role, maintain the parent-child role and address the strain experienced by the whole family. Equally, enabling young carers to spend time with peers and participate in activities outside the home may help them in developing their identity outside of being a carer. This further supports calls across Europe for a greater emphasis on understanding the young carer as a child first and foremost, and a focus on considering the needs of the care recipient within the context of the wider family and social care system.^6,73^

Healthcare professionals may also be able to support young carers with accessing wider services. Young carers described often being unaware of services and the lack of signposting from professionals with whom they were in contact. Healthcare professionals can help improve this signposting by ensuring that they are comprehensive in their assessment of young carers’ needs (through evidence-based interventions such as CSNAT-I), providing appropriate information about services to young carers, making referrals on their behalf and supporting them to engage with the services they require. The review highlighted a particular need to support young carers within minority ethnic communities in accessing services.

Finally schools play a key role in identifying young carers, enabling them to manage the dual respsonsibilities of academic work and their caring role, and encouraging and promoting participation in a range of social opportunities and activities. The review identified, however, that being a young carer can result in absenteeism and poor academic performance. Future education plans were also impacted by caring responsibilities. The review’s findings reflect wider understanding of the need to promote and enable young carers’ learning and educational opportunities, together with supporting their school engagement and attendance.^74^ Healthcare professionals who work in schools can help young carers to consider the full range of their physical, emotional and social needs, as well as liaise with educational staff about how young carers might balance being able to fully participate in their education, and the other opportunities offered via school, with being a carer.^71,75^

In summary, a major finding of the review is the range of unmet support needs among young carers. This is supported by wider research and a number of reports produced by organisations that seek to advocate for, and support, young carers.^1,2,10,14,15,19–21,76^ Further, the United Nations Convention on the Rights of the Child sets out the civil, political, economic, social and cultural rights of every child (regardless of race, religion or abilities), requiring governments to meet children’s basic needs, help them reach their full potential and have a right to a family life.^77^ It is therefore likely that young carers would benefit from on-going conversations to identify their unmet support needs enabling appropriate and tailored support from health and social care professionals. Using the CSNAT Intervention to identify support needs and develop an action plan to meet these needs may be one way of accomplishing this although, as we now discuss, the review also indicates that the CSNAT itself (the tool underpinning CSNAT-I) would benefit from adaptation for young carers.

#### Comprehensiveness of the CSNAT v3.0 for young carers

The questions on CSNAT v3.0 covered most of the young carers’ support needs identified by the review, and no question was redundant. However, based on the findings of this review, adaptations could be made to enhance the comprehensiveness of CSNAT v3.0 for young carers in relation to their educational needs and parent-child relationships. This is unsurprising given that previous CSNAT work (in both its development and testing) related only to the support needs of adult carers.

The explicit inclusion of need for support with ‘education’ on the CSNAT could help open conversations with young carers about their individual support needs related to this and ways to address them. Discussion with one of the two original CSNAT developers concluded that, for young carers, the adaption of question 4 on CSNAT v3.0 (‘Do you need more support with your financial, legal or work issues?’) to include ‘educational issues’ might address this, that is, ‘Do you need more suport with your educational, financial, legal or work issues?’.

Although CSNAT v3.0 includes a question on ‘managing relationships’ (question 7), the centrality, complexity and range of impacts of the parent-child relationship for/on young carers suggests it warrants a new, separate, dedicated CSNAT question of its own for young carers. However, the addition of a new parent-child relationship question would require changes to the existing ’managing relationships’ question (question 7) in two ways: (1) the addition of the word ‘other’ to its current wording that is, ‘managing other relationships’ and (2) re-locating this question to follow the new parent-child relationship question (so that the inclusion of ‘other’ is logical). Consultation with one of the two original CSNAT developers confirmed this.

### Strengths and limitations

To our knowledge this is the first review to explore the comprehensiveness of the well-established internationally-adopted CSNAT for use with young carers. The review benefitted from the expertise and insight of one of the two original CSNAT developers. Included papers were exclusively qualitative: the use of qualitative methods in these studies yielded a wealth of information such that ‘support needs’ could be identified within their narratives.^39,40,56,60,66^

This review has some limitations resulting from limited resources. Excluding grey literature and papers not written in English language may have resulted in the loss of potentially useful information. Including grey literature can increase the quality of a review, as well as reduce publication bias.^78^ Authors of papers included in the review were not contacted as field experts: such an approach might have identified additional literature not captured by the searches. Further, search terms were limited to title field only (due to the excessive hits resulting from full paper or abstract searches), however citation searching was used as a supplementary strategy.

The population subject of this review, young carers, are challenging to identify. Some of the reviewed papers commented that young carers are a ‘hard to reach population’^51,56^ and this is supported by the broader young carers’ literature.^79–81^ Moreover, young carers’ concerns about the associated stigma of the ‘young carer’ label might limit the representativeness of study samples to the population. Likewise, some papers indicated young carers’ worries related to privacy invasion and unwanted interventions for their families.^81,82^ It is worth noting that the participants in some of the included studies were not labelled as ‘young carers’, instead being described as ‘young people’, ‘children’ or ‘adolescents’.^38,40,56,60,66^

Finally, the process of mapping support needs to CSNAT v3.0 was, to a certain degree, subjective due to both the complexity and the nature of ‘support needs’. It was also conducted by adult researchers. Young carers may have different interpretations of the CSNAT questions compared to adult researchers. The proposed adaptations to CSNAT v3.0 are an attempt to enhance the comprehensiveness of the tool for this group, to ensure it includes the full range of domains of support needs for young carers in order to prompt them to consider and identify their individual support needs. Young carers should be engaged in the actioning of the suggested CSNAT adaptions to ensure appropriate wording and relevance, then validation, but this was beyond the scope of this review.

Establishing the acceptability of a CSNAT which includes the proposed adaptations for young carers is also beyond the scope of this review, as is establishing the acceptability and feasibilty of CSNAT-I (the approach, as opposed to the tool itself) for young carers. Exploring both of these with young carers, alongside implementation work with health, social care and education professionals, are key next steps.

### What this review adds?

This review identifies and synthesises the support needs of young carers, as identified by young carers themselves. It explores the comprehensiveness of the well-established, internationally used, CSNAT for use with carers of a younger age than previously engaged with. The findings suggest that CSNAT-I, using the adapted CSNAT for young carers, could be a useful means for health, social care and education professionals to work with young carers in order to identify and address their support needs.

## Conclusion

The findings of this review highlight that young carers have a range of support needs arising from their caring role, but that many of these needs remain unmet. Delivery of the evidence-based CSNAT Intervention by appropriately trained and skilled professionals could address this, but this review identified that the CSNAT (the tool underpinning CSNAT-I) requires adaptation in order to include questions related to ‘education’ and the ‘parent-child relationship’, thereby enhancing its comprehensiveness for young carers.

## Supplemental Material

sj-docx-1-pmj-10.1177_02692163251363476 – Supplemental material for Does the Carer Support Needs Assessment Tool (CSNAT) cover the support needs of young carers? A systematic literature search and narrative review Supplemental material, sj-docx-1-pmj-10.1177_02692163251363476 for Does the Carer Support Needs Assessment Tool (CSNAT) cover the support needs of young carers? A systematic literature search and narrative review by Yuen Ki Fung, A Carole Gardener and Morag Farquhar in Palliative Medicine
