# Supplementary material for: Does the Carer Support Needs Assessment Tool (CSNAT) cover the support needs of young carers? A systematic literature search and narrative review
Source: Palliat Med. 2025 Aug 25;39(9):965–76. doi: 10.1177/02692163251363476 (PMC13044424; doi:10.1177/02692163251363476)
Supplement: sj-docx-1-pmj-10.1177_02692163251363476 – Supplemental material for Does the Carer Support Needs Assessment Tool (CSNAT) cover the support needs of young carers? A systematic literature search and narrative review [file sj-docx-1-pmj-10.1177_02692163251363476.docx]

| **Appendix 1: Characteristics of included studies** | | | | | | |
| --- | --- | --- | --- | --- | --- | --- |
| **Study** | **Aim** | **Design & data collection** | **Recruitment** | **Analysis** | **Characteristics of young carers** | **Characteristics of those being cared for** |
| Addo et al, 2021^61^  (Australia) | To examine the effects of  caring on the lives of young carers in New South Wales and their support-seeking behaviour | Qualitative: semi-structured interviews/focus groups | Fliers in schools and universities  Fliers/emails via young carers’ organisations  Snowballing | Identification of codes/themes | N=28 young carers  Female (n=23)  Male (n=5)  Age 16–26 years (data only extracted for carers aged 18yrs or younger) | No information provided |
| Andersen, 2012^62^  (Western Kenya) | To highlight the strategies that children adopt in  order to cope with the hardship they face when becoming  a primary caregiver to an HIV-infected parent on antiretroviral therapy in Western Kenya | Qualitative: ethnographic data collected through in-depth interviews, focus group discussions, drama, diaries and participant observation | Identified by local community  health workers | Followed Emerson’s (1995) five phases of analysis: reading, open coding, focused coding, initial memos, and integrative memo | N= 20 young carers  Age 6-16 years | HIV-infected parent on antiretroviral therapy |
| Barry, 2011^63^  (UK: Scotland) | To explore the views and experiences of young carers in Scotland about their social  networks and experiences of relationships with others, such as family, friends  and teachers | Semi-structured interviews | Via young carers’ projects | No information provided | N= 20 young carers  Female (n=10)  Male (n=10)  Age 12–23 years (data only extracted for the n=18 carers aged 18 years or younger) | Mental or physical disabilities, mental  health problems, ADHD, alcoholism and physical illnesses. Majority of the young carers were looking after their mother (although both  parents ill or disabled in two cases); some were looking after siblings with disabilities or  ADHD |
| Blake-Holmes & Cook, 2024^64^  (UK) | To report young carers’ retrospective accounts of professional support when caring for a parent with mental illness during childhood | Qualitative: biographical narrative interviews | Researcher speaking at public events, such as Mind, Young Carers’ conferences and through social media | Thematic narrative method | N=20  Female (n=15)  Male (n=5)  Age 19-54 years  (former young carers reflecting back on their childhood experiences of being a young carer) | Parents with mental ill health |
| Brimblecombe et al, 2024^65^  (UK)  *Sits within the same study as Stevens et al 2024* | To provide new knowledge and address prior research gaps regarding how best to support young carers  from their perspective and that of the people they care for | In-depth qualitative methodology using focus groups, in-depth semi-structured  interviews and workshops in four localities in England | Young carers’ organisations in four localities in England with contrasting ethnicity and rurality, and including areas of high deprivation | Thematic analysis | N=133  Age 9–25 years  Including:  N=41 aged 9-11  N=57 aged 12-15  N=36 aged 16-25  (data only extracted for carers aged 18 years or younger) | Where known, 25 cared for a sibling, 46 for a parent and 2 for another  relative. Some cared for more than one person. Reasons for needing care, where known, were mental ill  health (N=26), physical ill health (N=19), substance misuse (N=8) and other (N=5: e.g.,  dementia, neurodiversity and learning disability). Many had mental and physical ill health needs. |
| Charles et al, 2011^40^  (Canada) | To explore caregiving experiences of young people from immigrant families and how the caring experience impacted their lives | Qualitative sub-study of young carers within British Columbia study: semi-structured interviews | Distributed posters in a broad range of locations | Thematic analysis | N=20 young carers  Female (n=17)  Male (n=3)  Age range: participants were adults who were young carers when 8-17 years old | No information provided |
| Cluver et al, 2011^66^  (South Africa) | To determine educational impacts of household AIDS-sickness  and other sickness among adolescent young carers | Phenomenological  qualitative approach: in-depth, open-ended qualitative interviews (including drawings) | Door-to-door sampling in 20 rural communities or urban townships identified as the most deprived in the Western Cape, and through schools | Thematic  content analysis, followed by a phenomenological approach to analysis for the  experiences of young people caring for sick relatives | N=659 young carers  Age range: 10-20yrs (data only extracted for carers aged 18 years or younger) | AIDS |
| D’Amen et al, 2022^67^  (Italy)  *Sits within the same study as Santini et al 2022* | To describe the experiences of Italian adolescent young carers  of grandparents,  to explore difficulties encountered and support needed in the caring role | Qualitative data drawn from an online survey  within EU Horizon 2020 Me–We project. Two open-ended questions focused on (1) help that would support the young carers and (2) main difficulties encountered as a young carer | Europe-wide convenience sample; Italian respondents  mainly recruited among high school students from a region in the north and a region in centre of Italy | Open coding process, followed by grouping of codes referring to same phenomenon  into sub-themes, and then higher-order themes, finally described in conceptual maps | N=87 young carers  Female (n=58)  Male (n=27)  Transgender/non-binary (n=2)  Age 15-17 years | Grandparents (older adults aged 65 years and over) with mainly from physical disabilities  (62.1%) or cognitive impairments (35.6%) |
| Doutre et al, 2013^68^  (UK) | To explore young carers’ experiences to seek to better understand their lives through a strength-based perspective | Qualitative: Semi-structured interviews and photo elicitation techniques | Recruited through a Young Carers Project in a rural area of England | Interpretive Phenomenological  Analysis | N=6 young carers  Age 11-13 years | Mental illness |
| Gowen et al, 2022^69^  (UK) | To inform understanding of children's entitlements, societal obligations towards them and implementation of the legislation in terms of ethical and legal boundaries of children's caring responsibilities | Qualitative (“interpretive”): focus groups | Service users of a young carers project in the north of England | Thematic analysis | N=21 young carers  Age 9-18 years | Fibromyalgia, anxiety and depression, autism, visual impairment, mental health diagnosis, substance abuse, learning disability, physical disability |
| Hamilton & Adamson, 2013^43^  (Australia) | To explore the caring responsibilities, impacts and needs of young carers | Qualitative: semi-structured interviews plus short online questionnaire post- interview | Via partner organisations displaying posters at educational institutions and health service providers | Thematic analysis | N=23 young carers  Female (n=8)  Male (n=14)  Age range: 8-17 years  N=13 young adult carers  Age range: 18-25 years (data only extracted for carers aged 18 years or younger) | Physical health (n=15)  Mental health (n=13)  Intellectual (n=10)  Long term condition (n=9)  Mother (n=9)  Father (n=6)  Siblings or other relatives (n=15) |
| Janes, 2022^70^  (UK) | To identify the impacts of the caring role across young carers who access or chose not to access support | Qualitative | Via schools and young carers projects | Phenomenological approach | N=10  Female (n=6)  Male (n=4)  Age 11-16 years | Mother (n=8)  Sibling (n=2)  Grandfather and mother (n=1)  Bi-polar (n=3); diabetes and hearing loss (n=1); multiple sclerosis (n=1); spina bifida,  hydrocephalus, and  epilepsy (n=1); aplastic anaemia (n=1); autism (n=1); cerebral palsy and learning difficulties (n=1); old age, dizziness, confusion (n=1); former substance misuse and mental health (n=1) |
| Kaiser et al, 2014^71^  (Germany) | To explore how schools can develop comprehensive, sustainable  support systems for young carers | Secondary analysis of an existing data set | No information provided | Qualitative analysis involving coding and categorisation undertaken using MAXQDA | N=9  Age 13-16 years | No information provided |
| Leu et al, 2018^47^  (Switzerland) | To explore the personal experiences and perceptions of young carers | Qualitative: semi-structured interviews and semi structured questionnaire with open questions | Healthcare services, schools and school based social workers, social services and NGOs working with specific illness types (including palliative care associations, home care nursing services and others) | Thematic analysis | N=15 young carers  Female (n=11)  Male (n=4)  Age under 18 years  N=14 young adult carers  Female (n=13)  Male (n=1)  Age range: 18–25 years (data only extracted for carers aged 18 years or younger) | Physical health (n=11)  Mental health (n=1)  Addiction (n=1)  No diagnosis (n=2)  Mother (n=6)  Father (n=1)  Siblings (n=8) |
| Matzka & Nagl-Cupal, 2020^72^  (Austria) | To identify psychosocial resources used by young carers in Austria | Qualitative descriptive study: photo elicitation interviews | Via private and  state‐funded healthcare and service providers operating throughout Austria that may have encounters with young carers | Directed qualitative content analysis and using the theoretical lens of resilience | N=10 young carers  Female (n=4)  Male (n=6)  Age range: 9-17 years | Chronically ill siblings or parents |
| Mauseth & Hjälmhult, 2016^45^  (Norway) | To identify the main concerns and coping strategies of young carers who care for their parents with multiple sclerosis | Qualitative: semi-structured interviews | Local Multiple Sclerosis Society and the Norwegian Multiple Sclerosis Competence Centre | Thematic analysis | N=15 young carers  Female (n=8)  Male (n=7)  Age range: 12-18 years | Permanent wheelchair user (n=3)  Using walking aid (n=7)  Mother (n=8)  Father(n=7) |
| McAndrew et al, 2012^36^  (UK) | To report on a participatory project which aimed to understand the needs of young carers | Participatory qualitative research involving participation in a young carer support group (VOCAL) presentation | Hosting of two World Café-based events to raise awareness of the mental well-being of young people, initially within the local community | Thematic analysis | N=6 young carers  Age range: 13-17 years | No information provided |
| McDougall et al, 2018^73^  (Western Australia) | To explore the lived experiences of young carers | Qualitative descriptive design using a phenomenological framework through in-depth semi-structured interviews | Via online platforms and through posters in educational  institutions, health service providers, carer and youth support service  offices, shopping centres, libraries and recreation centres | Thematic analysis | N=13 young carers  Age range: 14-25 years (data only extracted for carers aged 18yrs or younger) | Physical disabilities (e.g. cerebral palsy), a chronic life limiting or terminal illness (e.g. dementia or advanced cancer), or mental health problems (e.g. schizophrenia) |
| McGibbon et al, 2019^74^  (UK: Northern Ireland) | To identify the factors which contributed to, or challenged, the resilience of young carers | In-depth semi-structured interviews | Via Barnardo’s Young Carers and Action for Children Young Carers Projects, along with the Health and Social Care Board | Thematic analysis | N=22 young carers  Female (n=18)  Male (n=4)  Age range: 8-18 years | Adult care recipients had a combination of mental and physical health problems. Care recipients under eighteen were living with conditions including cerebral  palsy, ADHD and autism. N=11 cared for more than one person. A third reported childcare responsibilities whilst providing care and support for one or more care recipients. |
| Moore et al, 2011^44^ (Australia) | To investigate the caring role and impacts of caring for a relative with alcohol or drug issues | Exploratory qualitative research project: open-end questions interview and prompts around themes developed from a literature review | Actively engaging agencies and services, and advertisements in newsletter articles and electronic bulletins | Thematic analysis | N=15 young carers  Female (n=8)  Male (n=7)  Age range: 11-17 years  N=12 participated in interviews  N=3 participated in focus group | Alcohol or drug issues (many were poly-drug users)  Mother (n=8)  Father (n=5)  Sibling (n=2) |
| Mordoch,  2010^37^  (UK) | To explore how children understand parental mental illness and the message they want to tell other children | Qualitative: secondary analysis of a grounded theory study | Original study (Murdoch & Hall, 2008): mental health professionals working in acute care, community and consumer support groups distributed letters to parents explaining the study. Also, posters and adverts placed in newsletters and a community newspaper | Thematic analysis (secondary) | N=22 young carers  Female (n=8)  Male (n=14)  Age range: 6-12 years | Primary diagnosis of depression, bipolar disorder or schizophrenia and receiving treatment |
| Newman et al, 2019^41^  (Canada) | To assess young carers’ experiences and needs of caring for a relative living with dementia | Qualitative (retrospective) descriptive study: semi-structured-individual interviews conducted by telephone or Skype | Youth organizations, social media and a youth dementia awareness symposium held at the primary author’s university | Thematic analysis | N=5 young carers  Female (n=5)  Age range: adults who were young carers when 12–18 years old | Dementia (n=5)  Grandmother (n=2) Grandfather (n=2)  Mother (n=1) |
| Nicholls et al, 2017^75^  (UK) | Study 1 of two studies reported in the paper: To determine whether current knowledge from cancer literature regarding young carers is generalisable to chronic conditions and, therefore, whether an existing screening tool could be adapted for this population | Study 1 of two studies reported in the paper: qualitative interviews | Study 1 of two studies reported in the paper: opportunistic sampling from UK colleges | Study 1 of two studies reported in the paper: interpretive phenomenologcal analysis (Study 1 of two studies reported in the paper) | Study 1 of two studies reported in the paper:  N=7 young carers  Age range: 17-19 years (data only extracted for carers aged 18yrs or younger: n=6) | Study 1 of two studies reported in the paper: cared for parents lived with migraine, arthritis, epilepsy, renal failure/kidney disease, myalgic encephalopathy |
| Nichols et al, 2013^49^  (USA & Canada) | To understand the needs and caring experiences of young carers who care for people with frontotemporal dementia (FTD) to create a relevant support website for young caregivers of dementia patients | Qualitative: semi-structured interviews | Young carers (from US and Canada) on a list of parents of young carers of people affected by FTD who were interested in voicing their opinions | Thematic analysis | N=14 young carers  Age range: 8-18 years  Focus group 1: (n=6)  Focus group 2: (n=8) | Frontotemporal dementia  Mother (n=2)  Father (n=10) (includes two stepfathers) Grandfather (n=2) |
| O’Dell et al, 2010^38^  (UK) | To explore young peoples’ thoughts on providing informal care for relatives | Qualitative: in-depth interviews using vignettes | Young carers from six schools and colleges in South East and south coast of England | Thematic analysis | N=46 young carers  Age range: 15-18 years | No information provided |
| Phelps, 2021^76^  (UK) | In relation to Hampshire Young Carers Alliance to explore: (a) what are the most important changes that the young carer services made to young carers and their families? (b) what is it about the services that creates those changes? | Qualitative: Semi-structured interviews | Via young carer projects in the Hampshire Young Carers Alliance | Thematic analysis | N=8  Age 9-17 years | No information provided |
| Santini et al, 2022^77^  (Italy and Slovenia)  *Sits within the same study as D’Amen et al 2022* | To shed a light on the needs and difficulties faced by a sample of adolescents aged 15-17, caring for grandparents living in Italy and Slovenia | Qualitative | Via secondary schools in Italy and Slovenia | Open coding process to identify concepts and their dimensions within the data with the support of MAXQDA | N= 162 living in Italy (n=87) and Slovenia (n=75)  Age 15–17 years | Grandparents  Physical Disabilities (n=102); cognitive Impairments (n=50); mental health (n=36); addiction (n=12); other (n=38) |
| Stamatopoulos, 2018^42^  (Canada) | To explore the positive and negative impacts for young carers delivering unpaid familial caregiving | Qualitative: two focus groups and one in-depth interview, plus short survey post- discussion | Via formal young carers programs e.g., Young Carers Program (YCP) in Toronto and the Powerhouse Project’s Young Carers Initiative (YCI) in the Niagara-Haldimand region | Thematic analysis | N=15 young carers  Age range:15-18 years  Focus group 1: Toronto (n=10)  Focus group 2: Niagara region (n=4)  One interview with a young carer who had previously received program support | Conditions: substance (alcohol) abuse, terminal cancer, autism, and high likelihood of comorbidity  Relationship: >50% were siblings; next largest group parents; several provided care for multiple family members |
| Stevens et al, 2024^78^  (UK)  *Sits within the same study as Brimblecombe et al 2024* | To explore the experiences and views of young carers on aspects of services and support seen as helpful, valued, and acceptable, and what could be improved | Qualitative: focus groups or interviews | Young carers organisations in four localities in England with contrasting ethnicity and rurality, and including areas of high deprivation | Reflexive thematic analysis | N=133 young carers  Aged 9–25 years (data only extracted for carers aged 18 years or younger) | Parents, siblings or other relatives with mental ill health (n=26), physical ill health (n=19), substance misuse (n=8) or other (including dementia, neurodiversity, learning disability) n=5) |
| Svanberg et al, 2010^39^  (UK) | To discover children’s experience and coping strategies, and how caring responsibilities impact their lives | Qualitative: semi-structured interviews, plus three quantitative measures | Via four UK dementia charities or an advertisement | Thematic analysis | N=12 young carers:  Female (n=6)  Male (n=6)  Age range: 11-17 years | Dementia    Female (n=2)  Male (n=7) |
| Szafran et al, 2016^79^  (Canada) | To explore the experiences of young carers from the perspective of former young carers | Descriptive, exploratory study using focus groups | Purposefully recruited through community agencies using a recruitment  notice, and snowballing | Descriptive perspective generating themes | N=5 former young carers  Age range: participants were adults who were young carers when under 18 years old | Parents or siblings living with schizophrenia, osteoarthritis, alcoholism, or autism, or abusive or neglectful parents |
| Trondsen, 2012^46^ (Norway) | To explore the lived experience of young people living with mentally ill parents and potential role of online self-help groups for adolescents with mentally ill parents | Qualitative, action-oriented study: analysis of the conversation in an online self-help group for adolescents with mentally ill parents (about 600 messages) | Users from a Norwegian hospital website (comprising information pages and open access forum) for children and adolescents with mentally ill parents | Thematic analysis | N=16 young carers  Female (n=15)  Male (n=1)  Age range: 15-18 years | Conditions: bipolar disorder, some had had psychotic episodes or had attempted suicide |
| Van Parys & Rober, 2013^48^ (Belgium) | To discover children’s experience of parental depression and their caring roles in the family | Qualitative: semi-structured family interviews | Belgian university hospital psychiatric unit | Thematic analysis (one theme used as microanalysis in one family interview) | N=14 young carers  Female (n=9)  Male (n=5) | Depression  Mother (n=7)  Father (n=1) |
| Yuan & Ku, 2024^80^  (Taiwan) | To listen to the voices of former young carers and explore their sense of responsibility, their response to family and societal expectations and how these affected their lives | Qualitative: interviews | Potential participants were recruited through advertisements in mental health and social services of Taiwan, and through social media | Thematic analysis | N=19  Female (n=11)  Male (n=8)  Aged over 18 years  (Former young carers reflecting back on their childhood experiences of being a young carer) | Parents    Schizophrenia and bipolar disorder (n=1); schizophrenia (n=9); chronic mental illness (n=2); anxiety disorder (n=1); depression (n=1); bipolar disorder (n= 4); affective disorder (n=1) |

| **Appendix 2: Overview of Critical Appraisal Outcomes (using Critical Appraisal Skills Programme Qualitative Checklist)^33^** | | | | | | | | | | | |
| --- | --- | --- | --- | --- | --- | --- | --- | --- | --- | --- | --- |
|  | **Was there a clear statement of the aims of the research?** | **Is a qualitative methodology appropriate?** | **Was the research design appropriate ‘to address the aims of the research?** | **Was the recruitment strategy appropriate to the aims of the research?** | **Was the data collected in a way that addressed the research issue?** | **Has the relationship between researcher and participant been adequately considered** | **Have ethical issues been taken into consideration?** | **Was the data analysis sufficiently rigorous?** | **Is there a clear statement of findings** | **How valuable is the research?** | **Total score** |
| Addo et al, (2021)^61^ | Yes | Yes | Yes | Yes | Yes | Yes | Yes | Yes | Yes | Yes | 10/10 |
| Andersen (2012)^62^ | Yes | Yes | Yes | Yes | Yes | Yes | Yes | Yes | Yes | Yes | 10/10 |
| Barry (2011)^63^ | Yes | Yes | Yes | Yes | Yes | No | Yes | Can’t Tell | Yes | Yes | 8/10 |
| Blake-Holmes & Cook (2024)^64^ | Yes | Yes | Yes | Yes | Yes | No | Yes | Yes | Yes | Yes | 9/10 |
| Brimblecombe et al (2024)^65^ | Yes | Yes | Yes | Yes | Yes | Yes | Yes | Yes | Yes | Yes | 10/10 |
| Charles et al (2011) ^40^ | Yes | Yes | Can’t Tell | Yes | Yes | No | Yes | Can’t Tell | Can’t Tell | Yes | 6/10 |
| Cluver et al (2012)^66^ | Yes | Yes | Yes | Yes | Yes | Yes | Yes | Yes | Yes | Yes | 10/10 |
| D’Amen et al (2022)^67^ | Yes | Yes | Yes | Yes | Yes | No | Yes | Yes | Yes | Yes | 9/10 |
| Doutre et al (2013)^68^ | Yes | Yes | Yes | Can’t Tell | Yes | Yes | Can’t Tell | Yes | Yes | Yes | 8/10 |
| Gowen et al (2022) | Yes | Yes | Yes | Yes | Yes | Yes | Yes | Yes | Yes | Yes | 10/10 |
| Hamilton & Adamson (2013) ^43^ | Yes | Yes | Yes | Yes | Yes | No | Yes | Can’t Tell | Yes | Yes | 8/10 |
| Janes (2022)^70^ | Yes | Yes | Yes | Yes | Yes | Yes | Yes | Yes | Yes | Yes | 10/10 |
| Kaiser et al (2024)^71^ | Yes | Yes | Yes | Can’t Tell | Yes | Yes | Yes | Yes | Yes | Yes | 9/10 |
| Leu et al  (2018) ^47^ | Yes | Yes | Yes | Yes | Yes | Yes | Yes | Yes | Yes | Yes | 10/10 |
| Matzka & Nagl-Cupal (2020)^72^ | Yes | Yes | Yes | Yes | Yes | No | Yes | Yes | Yes | Yes | 9/10 |
| Mauseth & Hjälmhult (2016) ^45^ | Yes | Yes | Yes | Yes | Yes | No | Yes | Yes | Yes | Yes | 9/10 |
| McAndrew et al (2012) ^36^ | Yes | Yes | Yes | No | Yes | No | Can’t Tell | Yes | Yes | Yes | 7/10 |
| McDougall et al (2018)^73^ | Yes | Yes | Yes | Yes | Yes | No | Yes | Yes | Yes | Yes | 9/10 |
| McGibbon et al (2019)^74^ | Yes | Yes | Yes | Yes | Yes | No | Yes | Yes | Yes | Yes | 9/10 |
| Moore et al (2011) ^44^ | Yes | Yes | Yes | Yes | Yes | No | Yes | Yes | Yes | Yes | 9/10 |
| Mordoch  (2010) ^37^ | Yes | Yes | Yes | Yes | Yes | Can’t Tell | Yes | Yes | Yes | Yes | 9/10 |
| Newman et al (2019) ^41^ | Yes | Yes | Can’t Tell | Yes | Yes | No | Yes | Yes | Yes | Yes | 8/10 |
| Nicholls et al (2017)^75^ | Yes | Yes | Yes | Can’t Tell | Yes | No | Yes | Yes | Yes | Yes | 8/10 |
| Nichols et al (2013) ^49^ | Yes | Yes | Yes | Yes | Yes | Yes | Yes | Yes | Yes | Yes | 10/10 |
| O’Dell et al (2010) ^38^ | Yes | Yes | Yes | Yes | Yes | No | Yes | Yes | Yes | Yes | 9/10 |
| Phelps (2021)^76^ | Yes | Yes | Yes | Yes | Yes | No | Yes | Yes | Yes | Yes | 9/10 |
| Santini et al (2022)^77^ | Yes | Yes | Yes | Yes | Yes | No | Yes | Yes | Yes | Yes | 9/10 |
| Stamatopoulos (2018) ^42^ | Yes | Yes | Yes | Yes | Yes | No | Yes | Can’t Tell | Yes | No | 7/10 |
| Stevens et al (2024) | Yes | Yes | Yes | Yes | Yes | Yes | Yes | Yes | Yes | Yes | 10/10 |
| Svanberg et al (2010) ^39^ | Yes | Yes | Yes | Yes | Yes | Yes | Yes | Yes | Yes | Yes | 10/10 |
| Szafran et al (2016)^79^ | Yes | Yes | Yes | Yes | Yes | Can’t Tell | Yes | Yes | Yes | Yes | 9/10 |
| Trondsen (2012) ^46^ | Yes | Yes | Yes | Yes | Yes | Yes | Yes | Yes | Yes | Yes | 10/10 |
| Van Parys & Rober (2013) ^48^ | Yes | Yes | Can’t Tell | Yes | Yes | Yes | Yes | Yes | Yes | Yes | 9/10 |
| Yuan & Ku (2024)^80^ | Yes | Yes | Yes | Yes | Yes | No | Yes | Yes | Yes | Yes | 9/10 |

| **Appendix 3: Mapping the findings of included papers to CSNAT-I v3.0 questions (domains of support need)** | | | |
| --- | --- | --- | --- |
| **CSNAT v3.0 Question** | **Unmet needs** | **Met needs** | **Helpful Input** |
| **1. Understanding your relative’s illness** | A need for information about the family member’s condition including the nature of the condition, the nature of treatment, potential complications and problems^36,37,41,45,48,49, 64,67,74,76-78^  Examples:   - Limited knowledge of the illness^41^ - Participants found it more difficult to access information about mental illness than other diseases^37^ - Young carers obtained information from overhearing adult conversations, causing worries^37^ *(also maps to CSNAT questions 6 and 7)* - Family members provided inconsistent or vague explanations to young carers^37,48^ - Sick parents would not proactively discuss their suicide attempts with participants (young carers) unless asked repeatedly^48^ *(also maps to CSNAT question 12)* - Young carers wanted more in-depth information about the illness, for example, the causes and how the disease would impact on relative^45^ - In some young carers’ families there was no discussion about the illness, which caused frustration^37,45,46^ *(also maps to CSNAT question 7)* - The doctor did not provide enough information^49^ - Young carers felt unimportant as health care professionals used professional terms and did not include them in decision making^36^ *(also maps to CSNAT questions 3 and 7)* | Received information from family members, the healthy parent and online searches^37,41,49^  Information from school counsellor and printed materials^37^ | Individualized approach (considering participant’s age and maturity) and using fun and creative way to disseminate the information^37,49^  A few of the participants found receiving age-related information through children’s books and verbal explanations by health care professionals helpful^45^  Participants did not wish to have more information about dementia but highlighted the importance of relative being honest about their problems to create meaning about why dementia affected their parent^39^ *(also maps to CSNAT question 7)*  It was important to be informed early about diagnosis thereby providing an explanation about the change in their parents^49^  Providing information can help reduce emotional distress and provide hope for the future^37^ *(also maps to CSNAT question 14)*  Providing information on medication and it’s usage^37^  Participants suggested that the school should implement public health promotions on aging and dementia to facilitate resources to become more accessible and reduce stigma^41^  Online forum would be an alternative way for young carer to seek help or share feelings and experience^49^ *(also maps to CSNAT questions 6 and 7)*  Young carer project groups provide a helpful source of information^76^  School is a source of information^66^ *(also links to education)* |
| **2. Having time for yourself in the day** | Caring is unexpectedly time consuming and tiring and led to a lack of time to complete assignments, plan for daily lives and leisure activities^41,42,46,74,79^  There was familial resistance to young carers having ‘alone time’^42^ *(also maps to CSNAT question 7)*  Young carers mentioned that caring responsibilities were time consuming that they did not have spare time to ‘idiotic things’^45^  Help finding opportunities to make friends or have a social life^36,39-43,45,47,70,71,80^ *(also maps to CSNAT question 7)*  Unable to participate in extracurricular activities or school groups due to afterschool caring responsibilities^42,79^ *(also links to education)*  Help organizing and dealing with multiple or excessive demands e.g., from school, caring etc.^64,67,70,76,77^ *(also links to education)* | Young carer asked little sister to cover their caring role to have time for oneself^36^  Role less time consuming due to parent taking more control of health, parents able to care for sibling at weekends, wider family involvement and young carer not being in the ‘frontline of care’^65,67,70^ *(also maps to CSNAT question 7)* | Young carer projects provide opportunities to be somewhere different to home, spend time with other people, have fun and relax^63,76^  Opportunity to delegate caring responsibilities to enable own leisure time^72^ |
| **3. Managing your relative’s symptoms, including giving medicines** | Understanding how to manage risk^44,77^ (e.g., required to continuously monitor sick parent’s symptoms to ensure parent’s safety^44^)  Unpredictable behaviours of sick parents were frightening^37^  Feelings of uncertainty about symptom management affected physical and emotional wellbeing^46^ *(also maps to CSNAT question 9)*  Worried about, and uncertainty identifying, parents’ suicidal thoughts ^46,48^  Accessing training and expert input^39,67,77^ (e.g., no training provided about how to manage parents’ dementia-related symptoms^39^)  Healthcare professionals used professional terms and didn’t fully explain changes in parent’s medication^36,37,49^ *(also maps to CSNAT questions 1 and 7)*  Young carers with parents with multiple sclerosis expressed difficulties in managing symptoms, such as fatigue and change in cognition, mood and responsiveness in a conversation^45^  Knowing how to monitor and manage symptoms, including managing difficult behaviour and motivating family members with mental health problems^37,44-46,48,67,70,77,80^  Knowing how to manage the cared-for person’s medication and therapy^36,67,77^ *(also maps to CSNAT question 6)*  Developing confidence in managing a crisis^67^ *(also maps to CSNAT question 6)*  Inputting into care plan meetings or aftercare arrangements following a parent’s discharge from hospital^64^ | The healthy parent educated the young carer in how to deal with the situation when the other parent was ill^37^  Able to manage the cared-for persons symptoms and the impact on the wider family through having a range of strategies^37,46,49^ *(also maps to CSNAT question 7)* | A good atmosphere at home would help to manage parent’s mental illness^46^  Being positive and realistic about the situation was helpful to relieve the impact of dementia to the family^49^ *(also maps to CSNAT question 7)*  Diabetes nurse provides support and information that led to a reduction in the young carer role^70^  Young carer groups provide information about how to manage the young carer role^76^  Involvement of professional carers  reduces young carer role^78^ |
| **4. Your financial, legal, or work issues** | Dealing with money, including managing financial instability^44,45,62,67,76^  Examples:   - Shoplifting food, father stealing money from young carer, and youth allowance being used for household expenditure illustrated need for more financial support^44^ - Worried about the financial stability of the family as only one parent had a job^45^   Necessary aids were expensive^45^ *(also maps to CSNAT question 10)*  Knowing what financial support is available^65,77,78^ and how to access it^65,67,78^  Needing to be self-sufficient and independent when encountering problems^38^ *(also maps to CSNAT question 6; also links to parent-child relationship)*  Overcoming disadvantages arising from caring role in relation to future job opportunities or education (e.g., missing out on work experience/education)^61,70,79^ *(also links to education)*  Not able to receive sufficient care and emotional support for parents caused low self-esteem, low confidence and feeling hopeless for the future^44^ *(also maps to CSNAT question 12; also links to parent-child relationship)* | Worked on occasional jobs in order to get what their friend’s parents bought for their children^45^  Care responsibilities viewed as an opportunity to explore career options in care-based professions^42,79^  Local business able to provide flexibility for young carers to have casual employment^42^  Gained skills related to work ethic, personal and community responsibility, understanding, sympathy, compassion, and independence^79^ | Information from social workers about accessing benefits^78^  Opportunities for flexible or casual employment^42,45^  Support with food and water from community health worker^62^  Money from school friends^62^ (*also maps to CSNAT question 7; also links to education)* |
| **5. Providing personal care for your relative (e.g., dressing, washing toileting)** | Help with moving and handling the cared-for person^67,77^  Dealing with unpleasant aspects of the physical care^67^  Help with complex aspects of physical care e.g., stoma bags^77^ | Home care nurses and the healthy parent would provide personal hygiene care for relative^45^ |  |
| **6. Dealing with your feelings and worries** | Providing care to others was the only way to provide personal value^40^  Both of the parents have less time to take care of young carers, so they tried to hide their feelings and emotions in order to cope^39^  Difficult to share caring experiences with friends due to caring role^39^ and stigma^41^ *(also maps to CSNAT question 7)*  Worried about friends’ reactions to sick parent’s symptoms and aids^42,45^ *(also maps to CSNAT question 7 and 10)*  Feeling overwhelmed balancing carer role and educational demands^42,46^  Difficulty managing temper when angry at school^42^  Struggling to maintain balance between being a child and a carer^49^  Parents’ needs were always young carers’ top priority and therefore young carers suppressed their needs and managed their emotions later^45^ *(also maps to CSNAT question 7; also links to parent-child relationship)*  Dealing with difficult feelings in relation to the cared-for person including frustration, anger, discomfort, sadness and guilt, elevated concerns, worry about providing adequate care and ensuring they are happy^38,39,42,44,45,48,66,67,69,70,74,76,77^  Examples:   - Feeling scared about observed changes in the care recipient’s condition^45,74^ - Feeling fear when alone with sick parents^45^ - Sick parents who previously had suicidal thoughts made young carers become extremely cautious about their behaviour^48^ - Feeling guilty when spending time with friends but not providing support for the sick parent^38^ - Elevated worries about care-recipient’s health condition and stability of the family^42^ *(also maps to CSNAT questions 7 and 14)* - The “young carer” label made young carers feel guilty about not doing enough^39^ (also maps to CSNAT question 7)   Overhearing family conversation caused worries^37^ *(also maps to CSNAT questions 1 and 7)*  Worried about inheriting mental illness from their sick parent^46^  Feeling powerless and confused when being prevented from helping sick parents^48^  Help managing a range of feelings including anger, stress, low mood, helplessness, concern about family dynamics, frustration and loneliness^67,70,76,79,80^  Managing the prolonged stress or feelings of burnout linked to providing long-term care^61,79^  Dealing with own mental health issues, including suicidal tendences, and self-harm^42,70^  Needing to be self-sufficient and independent when encountering problems^38^ *(also maps to CSNAT question 4; also links to parent-child relationship)*  Managing fears about losing parent due to their suicidal ideas or mental health symptoms^80^  Accessing professional support from mental health services and at school^67,77,78^ *(also links to education)*  Need for a trusted person to talk to, an online forum or a befriending service^39,41,44,49^  Needing opportunities for outside interests including listening to music, playing computer games, and doing activities with friends to help manage worries^45,46^  Help managing family dynamics with the aim of reducing the stress of the caring role^39,41,46,49^  Caring led to panic attacks and agitation^42^ *(also maps to CSNAT question 9)*  Knowing how to manage the cared-for person’s medication and therapy^36,67,77^ *(also maps to CSNAT question 3)*  Parents periodically incapable of participating in school or leisure activities with participants caused them felt sad^45,46^ *(also links to education and parent-child relationship)*  Developing confidence in managing a crisis^67^ *(also maps to CSNAT question 3)* | Family provided emotional support^41^  Counselling was useful^39^  Feeling good about sharing negative feelings with the other parent^49^ *(also links to parent-child relationship)*  Sharing worries with health care professionals could help boost self-confidence^46^ *(also maps to CSNAT question 7)*  A specific teacher and friends with whom the people young carers would share their worries with^41,48,63^ *(also maps to CSNAT question 8)*  Siblings provided nonverbal support, such as staying with them in the same bedroom^48^ *(also maps to CSNAT question 7)*  Sense of well-being (satisfaction  from caring role, happiness, confidence, self-worth, maturity, and independence) due to feeling supported^40,42,44,49,70^  Able to manage the role by learning to be patient and not stressing^61^  Able to cope due to strong relationship with the cared-for parent^61^ *(also links to parent child relationship)*  Able to cope by maintaining outside interests like sport^48,49^  Reduction in feelings of annoyance and frustration due to growing confidence in the role^70^  Better able to cope due to knowing that you are not alone and there are others with similar roles^76^  Less stress due to being able to share worries and concerns^78^  Able to share experiences of caring role with school friends^47^  Able to accept the chronically ill parent suffering physical and cognitive limitations and thus needing various aids^45^ *(also maps to CSNAT question 10)*  Feeling more confident about ability to carry out caring role resulting in a leading better balance between caring and school^70^ *(also links to education)* | Doing sports^48,49,72^  Finding someone (e.g., grandparents) outside the family for support, sharing negative feelings and worries with specific teachers and some friends as coping strategies^48,63^  Spending more time with the patient on revisiting old memories and doing activities that could help ease family conflicts and clarify mixed feelings about parents^49^  Listening to music, playing computer games, and doing activities with friends or other leisure activities were suggested as sources of respite and distraction from worries for young carers^45,46,72^  Detaching themselves from their parents’ relationship could be helpful in terms of feeling less responsible for caring and less upset^39,46^ *(also links to parent-child relationship)*  Talking to health care professionals would be helpful to release stress^46^  Befriending services and respite would be useful to relieve the burden on the family^39,44^ *(also maps to CSNAT question 7)*  Decreasing the interaction with the sick relative could help the alleviate the feelings of loss and sadness^41^ *(also maps to CNSAT question 7)*  Online forum would be an alternative way for young carer to seek help or share feelings and experience^49^ *(also maps to CSNAT question 1 and 7)*  Young carers found that the counselling service was helpful for them^39^ *(also maps to CSNAT question 8)*  One young carer invited their class home to show them the assistive equipment, then their understanding was a bit higher^47^ *(also maps to CSNAT questions 7 and 10)*  Accepting the situation might involve accepting the chronically ill parent suffering physical and cognitive limitations and thus needing various aids^45^ *(also maps to CSNAT question 10)*  Support from family or local young carers project^41,48,63,70,76,78^  Support from social services, counselling or young carers project that addresses young carers’ mental health issues^39,70,73^  Support from Special Educational Needs Coordinator (SENCo)^65,78^ *(also links to education)*  Informal support provided in school^61^ *(also links to education)*  Having trusted professionals to talk to who listen and understand^65,78^  Opportunities for fun friendships and relaxation within young carer groups^76^  “I’d like to talk” box at school^65,78^ *(also links to education)*  Good friends to confide in when it gets hard^77^ *(also maps to CSNAT question 7)*  School provides a distraction from caring role^66,72,79^  Being able to spend time alone to reflect on experiences and thoughts e.g., being able to retreat to own room^72^ |
| **7. Managing relationships** | Peer Relationships  Help overcoming difficulties in maintaining friendships due to prioritising caring role, feeling unable to confide in friends about role and disengagement from peer group interests ^39-42,45,46,64,70,71,73,74,78-80^  Examples:   - Had difficulty in building age-appropriate relationship due to the lack of opportunities^40^ - Often use school hours as an opportunity to socialize with friends leading to violation of classroom’s rules due to limited social hours after school^42^ *(also links to education)* - Friends lacked similarity and mutuality with young carers, so it was challenging to trust and talk to them about their situation^40,41,42,45,46^ - Caring responsibilities have affected participants’ relationship with the family and friends^41^ - Caring responsibilities created barriers to making friends^45^ - Difficulty in peer relationships due to caring role, stigma and loss of support from parents^39,41^ *(also maps to CSNAT question 6)*   Caring responsibilities affected young carers’ ability to participate in social activities^39,40,42,43,45,47^  Suffered isolation due to caring role taking up most of the time^36,40,42^  Frequently changing of schools because of family relocation^37^ *(also links to education)*  Worried about friends’ reactions to sick parent’s symptoms and aids^42,45^ *(also maps to CSNAT questions 6 and 10)*  Could not bring friends to home on sick parent’s ‘bad days’ and might need to cancel social activities^45^  Lack of support from parents and caring responsibilities accounted for young carers’ poor academic achievements and the difficulty in peer relationship^39^ *(also links to education and parent-child relationship)*  Frequently being bullied in school but could not reply on parents to deal with situation^36^ *(also links to parent-child relationship and education)*  Help finding opportunities to make friends or have a social life^36,39-43,45,47,70,71,80^ *(also maps to CSNAT question 2)* | Peer Relationships  Caring role manageable enabling young carer to socialise with friends^70^ | Peer Relationships  One young carer invited their class home to show them the assistive equipment, then their understanding was a bit higher^47^ *(also maps to CSNAT questions 6 and 10)*  Opportunities to share caring experience with friends^47,73^  Sharing things (financial need, feelings) with school friends^62^ *(also maps to CSNAT questions 4; also links to education)*  Fostering meaningful friendships^72^  Good friends to confide in when it gets hard^77^ *(also maps to CSNAT question 6)*  Friends providing ‘pseudo-parental support’ for young carers^42^ *(also links to parent-child relationship*)  Online forum would be an alternative way for young carer to seek help or share feelings and experience^49^ *(also maps to CSNAT questions 1 and 6)* |
|  | Familial Relationships  Managing the distribution of caring tasks within the family, including care of younger siblings and communicating with extended family ^39,42,44,45,47,64,67,70,74,77^  Examples:   - Feeling sense of unfairness that the family take young carer’s contribution forgranted^39,45^ - Fatigue and stress arising from having to act as a communication hub and repeatedly share updates to extended family^47^ - Lack of parental support to provide care for younger siblings^44^ - Unequal caring responsibilities worsened the familial relationship^42^   Parents needs were always top priority and therefore young carers always suppressed their needs and self-managed their emotions^45,49^ *(also maps to questions 6; also links to parent-child relationship)*  There was familial resistance to young carers having ‘alone time’ resulting in young carer sadness and frustration^46^  The “young carer” label made young carers feel guilty about not doing enough^39^ *(also maps to CSNAT question 6)*  Help dealing with the impact on family relations including lack of communication, feeling distant from family, jealousy towards siblings and family breakdown^37,40-42,44-47^  Examples:   - Frustration about the lack of discussion about the sick parent’s condition^37,45,46,47^ - Had nobody to talk to if the other parent was not available^45^ *(also maps to CSNAT question 8)* - Feeling distant from familial relationships^40^ - Feeling jealous because peers could interact with parents, but young carer could not interact with their parent^41,46^ - Elevated worries about the care-recipient’s health condition and the stability of the family^42^ (*also maps to CSNAT questions 6 and 14)* - Personality and behavioral change due to alcoholism and drug use caused frustration and family breakdown^44^ *(also links to parent-child relationship)*   The “young carer” label was not always helpful^39,73^  Managing difficulties communicating with, or understanding, the cared-for person^49,67,77^  Overhearing family conversation caused worries^37^ *(also maps to CSNAT questions 6 and 7)*  Help to transition from the carer role within the family to building an independent adult life^61,80^ | Familial Relationships  Feeling good about sharing negative feelings with the other parent^45^ *(also maps to CSNAT question 6)*  Siblings provided nonverbal support to young carers, such as staying with them in the same bedroom^48^ *(also maps to CSNAT question 6)*  The experience of being a carer made young carers feel they were closer to the family and facilitated their personal growth^42,44,49^  Role less time consuming due to parent taking more control of health, parents able to care for sibling at weekends, wider family involvement and young carer not being in the ‘frontline of care’^65,67,70^ *(also maps to CSNAT question 2)*  Feeling comfortable with the caregiving role due to family bonds/sense of family duty^67,80^  Able to manage the cared-for persons symptoms and the impact on the wider family through having a range of strategies^37,46,49^ *(also maps to CSNAT question 3)*  Felt trusted when relative willing to explain the situation to young carer^40^ *(also maps to CSNAT question 12)* | Familial Relationships  Support from, or bonding with, cared-for person, the healthy parent and the wider family^65,67,70,72,73^  Participants did not wish to have more information about dementia but highlighted the importance of relative being honest about their problems to create meaning about why dementia affected their parent^39^ *(also maps to CSNAT question 1)*  Small acts of gratitude from the cared for person^74^  Being positive and realistic about the situation was helpful to relieve the impact of dementia to the family^49^ *(also maps to CSNAT question 3)*  Befriending services and respite would be useful to relieve the burden on the family^39,44^ *(also maps to CSNAT question 6)*  Decreasing the interaction with the sick relative could help the alleviate the feelings of loss and sadness^41^ *(also maps to CNSAT question 6)* |
|  | Relationships with health and social care professionals  Young carers found that it was challenging to maintain the relationship with doctors or nurses who took care of their parents^41,49^  Being treated as invisible as nobody inside or outside the family, including clinicians, was willing to discuss the condition of mentally ill parents^46^  Being seen as unimportant because the clinicians used professional terms to explain sick parents’ condition and did not involve young carers in decision-making^36, 37,49^ *(also maps to CSNAT questions 1 and 3)*  Fear of government interference and lack of trust in social services^79^ | Relationships with health and social care professionals  Sharing worries with health care professionals could help boost self-confidence^46^ *(also maps to CSNAT question 6)*  Feeling reassured about sharing information with professionals if this is discussed with the young carer first^65^ | Relationships with health and social care professionals  Professionals working in a way that fits around the family^65^  Social workers providing information and help young carer access wider support^65,78^  Unscheduled visits by social workers providing useful ways of finding out what is really going on in the family^65,78^  Knowing which professional carer is coming in to look after the cared-for person^65^  Being able to talk to trusted professionals^65,78^ |
| **8. Knowing who to contact if you are concerned about your relative (for a range of needs including at night)** | Young carers mentioned that they were not aware that there were emotional or social support services available for them^41^  There is a need for a trusted person to help with practical tasks such as picking up siblings from school^36,44^  Feeling frustrated about the long wait for support services^36^  Difficult to obtain/inconsistency of support service system^36,37,39,46^  Had nobody to talk to if the other parent was not available for them^45^ *(also maps to CSNAT question 7)*  Struggled to find someone who would be willing to help without asking about the situation and difficulties^41^  Terminology was a barrier for young carer to access to support, as different agencies had various definitions of caregivers^41^  Needing someone to support them, however, no one ever called^40^  Moving away home became a difficult decision due to worries about the lack of someone to support the sick relative or increasing caring responsibilities for other family members^43^ *(also maps to CSNAT question 12)*  Access to expert advice^77^ | A specific teacher and friends with whom the people young carers would share their worries with^41,48,63^ *(also maps to CSNAT question 6)* | Young carers found that the counselling service was helpful for them^39^ *(also maps to CSNAT question 6)* |
| **9. Looking after your own health (physical problems)** | Dealing with symptoms such as excessive tiredness due to late nights or early mornings, excessive responsibilities, difficulty sleeping, panic attacks, stress and agitation^36,42,43,70^  Examples:   - Not sleeping well, feeling worried, stressed and depressed^43^ - No time to go to the GP and always feeling tired and negative about life^36^ - Caring led to panic attacks and agitation^42^ *(also maps to CSNAT question 6)*   Feelings of uncertainty about symptom management affected physical and emotional wellbeing^46^ *(also maps to CSNAT question 3)*  Making time to go to GP^36^ |  |  |
| **10. Equipment to help care for your relative** | Necessary aids were expensive^45^ *(also maps to CSNAT question 4)*  Some were unsure of how friends might react to their parents need for aids, of which some felt ashamed^45^ *(also maps to CSNAT questions 6 and 7)*  Accessing support via technology^49^ | Instrumental Activities of Daily Living performed by young carers included translation aid for parents who do not speak English^42^  Able to accept the chronically ill parent suffering physical and cognitive limitations and needing various aids^45^ *(also maps to CSNAT question 6)* | Accepting the situation might involve accepting the chronically ill parent suffering physical and cognitive limitations and thus needing various aids^45^ *(also maps to CSNAT question 6)*  Some young carers liked the idea of distant support and resources using technology^49^  One young carer invited their class home to show them the assistive equipment, then their understanding was a bit higher^47^ *(also maps to CSNAT questions 6 and 7)* |
| **11. Your beliefs or spiritual concerns** |  | Able to make peace, or feel resilience, in relation to loss^40,41^  Examples:   - One young carer described “making their peace” with the fact that their grandmother was no longer who she used to be and that they had lost her “mentally”: “I’ve made my peace with that but probably when I was 19 or 18 it was really hard to kind of make peace with [that]”^41^ - For some young carers, their most significant belief was that their caregiving experiences made them stronger people: “It made me stronger emotionally, because I’m able to go through the fact that I might lose a person. [I know] I have to be strong for that person”^40^ | Faith made some young carers perceive their caregiving positively e.g., made caring meaningful/purposeful and the children received blessings for their caring activities^62^ |
| **12. Talking with your relative about his or her illness** | Sick relatives would not disclose suicide attempts unless young carers explicitly, and repeatedly, asked about it^48^ *(also maps to CSNAT question 1)*  Participants felt jealous and sense of unfairness as their peers could interact with their family members, but they could not^41,46^  Feeling of being forgotten, not being able to rely on parents and feeling of missing out parent-child relationship^39^ *(also links to parent-child relationship)*  Not able to receive sufficient care and emotional support for parents caused low self-esteem, low confidence and feeling hopeless for the future^44^ *(also maps to CSNAT question 4; also links to parent-child relationship)*  Help talking to the cared-for person about caring role^78^ | Felt trusted when relative willing to explain the situation to young carer^40^ *(also maps to CSNAT question 7)* |  |
| **13. Practical help around the home or elsewhere** | Need for help with practical tasks and housekeeping^44,46^  Examples:   - Participants struggled by themselves with practical tasks^46^ - Need for assistance with housekeeping^44^ | Working with the healthy parent on practical tasks such as chores^39,49^  Sick parents’ personal hygiene was supported by home care nurse and the other parent^45^ |  |
| **14. Knowing what to expect in the future when caring for your relative** | Moving away home became a difficult decision due to worries about the lack of someone to support the sick relative or increasing caring responsibilities for other family members^43^ *(also maps to CSNAT question 8)*  Dealing with worries about the sick person’s prognosis, potential changes in caring responsibilities and future loss of the parent^37,43,48^  Examples:   - Worry about the prognosis of their sick parent and the change in caring responsibilities^43^ - Stressful feelings arising from not knowing what would happen to the sick parent in the future^37^ - Elevated worries about care-recipient’s health condition and stability of the family^42^ (also maps to CSNAT questions 6 and 7) - Existential fear about sick parent’s conditions because things would change when the sick parent died^48^ |  | Providing information about the illness can reduce emotional distress and provide hope for the future^37^ *(also maps to CSNAT question 1)* |
| **15. Getting a break from caring overnight** | Impact of providing care overnight because the care recipient was up all night^42^  Access to respite care^39^ |  | Participants suggested befriending or respite services would be useful for them to relieve their burden of the family^39^ |
|  | | | |
| **Areas of support need that could not be mapped to CSNAT v3.0 questions (domains)** | **Unmet Needs** | Meet Needs | **Helpful Input** |
| **Education** | Caring responsibilities had negative impacts on their education, such as occasional absences, decreased concentration during classes, not having enough time to complete schoolwork, some mentioned memory was affected together with their ability to complete schoolwork^37,39,42,46,47,61,62,66,70,78-80^  Postponed education due to caring responsibilities^42^  The choice of further education and courses were affected by care responsibilities^42,43,45^  Not able meet the 30-hours’ volunteer requirement for provincial graduation guidelines due to caring responsibilities^42^  Unable to participate in extracurricular activities or school groups due to afterschool caring responsibilities^42,79^ *(also maps to CSNAT question 2)*  Understanding and support from teachers about impact of young carer role^61,63,65,67,70,77,78^  Help dealing with experiencing, or seeing, stigma and bullying at school relating to disability and illness^36,71^ *(also maps to CSNAT question 7)*  Making time and space at home to do schoolwork^70,77^  Systems in schools to identify and support young carers^36^  Help organizing and dealing with multiple or excessive demands e.g., from school, caring etc.^64,67,70,76,77^ *(also* *maps to CSNAT question 2*)  Often use school hours as an opportunity to socialize with friends leading to violation of classroom’s rules due to limited social hours after school^42^ *(also* *maps to CSNAT question 7*)  Overcoming disadvantages arising from caring role in relation to future job opportunities or education (e.g., missing out on work experience/education)^61,70,79^ *(also maps to CSNAT question 4*)  Frequently changing of schools because of family relocation^37^ *(also maps to CSNAT question 7*)  Accessing professional support from mental health services and at school^67,77,78^ *(also maps to CSNAT question 6*)  Lack of support from parents and caring responsibilities accounted for young carers’ poor academic achievements and the difficulty in peer relationship^39^ *(also maps to CSNAT question 7; also links to parent-child relationship)*  Parents periodically incapable of participating in school or leisure activities with participants caused them felt sad^45,46^ *(also maps to CSNAT question 6; also links to parent-child relationship)* | Taking extra night school, summer school and online courses to improve grades^42^  Strengthened emotional bond with the parent due to caring role^44,45,48,49^  Caring role manageable so able to focus on schoolwork as necessary^70^  Care role acknowledged and taken into account re schoolwork^61^  Feeling more confident about ability to carry out caring role resulting in a leading better balance between caring and school^70^ *(also maps to CSNAT question 6)* | School teachers’ ability to identify young carers^36^  A checklist should be developed collaboratively with young carers to provide guidance for teachers or nurses on providing support for young carers^36^  Flexibility in education system (e.g., attendance and date of submission) would be useful for participants (young carers)^36,44^  Integrating the caring responsibilities into the young carers’ daily life, for example a young carer brought her grandfather with her to friends’ house to work on group project^41^  Teachers aware of carer role and need for support e.g., adjusting homework^61,63,71,78^  Being able to talk to trusted teachers^63,65,71,78^  Proactive support at school e.g., regular sessions with a member of the school staff to discuss issues^71,78^    Having a pass that enables young carer to leave school when cared-for person needs assistance^71,78^  Option to go to a quieter learning environment when feeling stressed or sad^71,78^  Peer education about disability and caring^71^  Support from young carer project led to young carer feeling better able to manage school^76^  Support from specialist role (SENCo/social worker) enabled young carer to better focus on schoolwork^65,78^ *(also maps to CSNAT question 6*)  Breakfast club^78^  “I’d like to talk” box at school^65,78^ *(also maps to CSNAT question 6*)  School is a source of information^66^ *(also maps to CSNAT question 1)*  Informal support provided in school^61^ *(also maps to CSNAT question 6*)  Support with caring role from other family members to enable school attendance^66^ |
| **Parent-child relationship** | Difficulty with peer relationships due to caring role and loss of support from parents^39,68^ *(also maps to CSNAT question 7)*  Parents periodically incapable of participating in school or leisure activities with participants caused them felt sad^45,46^ *(also maps to CSNAT question 6; also links to education)*  Personality and behaviour change in the sick relative (due to alcoholism and drug use) led to frustration and family breakdown^37^ *(also maps to CSNAT question 7)*  Lack of support from parents and caring responsibilities accounted for young carers’ poor academic achievements and the difficulty in peer relationship^39^ *(also maps to CSNAT question 7; also links to education)*  Managing difficult dynamics e.g., parent who overshares with young carer, young carer feeling they need to minimize parental guilt about impact on young carer, young carer protecting the parent from stigma, preventing parental distress, putting parents needs over their own, having to support parent’s work-life balance, child-parent role reversal^40,42,44,46,49,64-68,79^  Examples:   - Role reversal of young carers became ‘a parent’ of their parent which caused frustration and burden^40,42,68^ - Sick parents not able to fulfil their parental responsibilities for young carers^46^ - Young carer thought the relationship between her and her parent was like roommates rather than parent and child^44^   Dealing with the impact of having a sick parent e.g. not receiving sufficient care and emotional support, low self-esteem, lack of confidence, hopelessness, feeling forgotten, missing out on parent-child relationship, lack of childhood activities, lack of support with difficult situations, poor academic achievement, difficulties with peer-relationships, solving problems alone^36,38-41,44-46,49,66^  Examples:   - Dementia symptoms affected communication and meaningful interactions with the cared for relative^49^ - Frequently being bullied in school but could not reply on parents to deal with situation^36^ *(also maps to CSNAT question 7; also links to education)* - Feeling of being forgotten, not able to rely on parents and feeling of missing out parent-child relationship^39^ *(also maps to CSNAT question 12)* - Needing to be self-sufficient and independent when encountering problems^38^ *(also maps to CSNAT questions 4 and 6)* - Comparison with peers’ parent child-relationship highlighted feelings of lack of their own parent-child relationship^41^ - Feeling loss of parent when symptoms exacerbated^46^ - A young carer with a chronic illness wished her mother could provide support for her^41^ - Not able to receive sufficient care and emotional support for parents caused low self-esteem, low confidence and feeling hopeless for the future^44^ *(also maps to CSNAT questions 4 and 12)* - Feeling loss of childhood, helpless about the caring roles and taking on an adult role in the family^40^   Help for young carer to talk about their caring role with parents^65^  Parents’ needs were always young carers’ top priority and therefore young carers suppressed their needs and managed their emotions later^45^ *(also maps to CSNAT questions 6 and 7)*  Enabling opportunities for young carer to do ‘children things’ and be a child^64^ | The healthy parent provided sense of stability and emotional support for the family ^45,49^  Sick parents took good care of young carers and highlighted the mutual understanding and love between children and their parents^44,48^  Able to cope due to strong relationship with the cared-for parent^61^ *(also maps to CSNAT question 6*) | School should implement public health promotions on aging and dementia to reduce stigma^41^  Detaching themselves from their parents’ relationship could be helpful in terms of feeling less responsible for caring and less upset^39,46^ *(also maps to CSNAT question 6)*  Someone to talk to outside the family about their caring role^65^  Young carer projects provide an opportunity to be somewhere different to home, spend time with other people, have fun and relax^63,76^  Friends providing ‘pseudo-parental support’ for young carers^42^ *(also maps to CSNAT question 7*) |
